# Mesostructural fracture modeling of asphalt mixtures: Stochastic heterogeneity coupled with bilinear cohesive elements

**DOI:** 10.1371/journal.pone.0347555

**Published:** 2026-08-27

**Authors:** Meng Wan, Zhihui Liu

**Affiliations:** 1 China Merchants Chongqing Communications Technology Research & Design Institute Co., LTD., Chongqing, China; 2 Tibet Autonomous Region Transportation Comprehensive Administrative Law Enforcement Corps, Tibet, China; China Construction Fourth Engineering Division Corp. Ltd, CHINA

## Abstract

Under the action of traffic loads, asphalt mixtures will crack to different degrees, which directly affects the serviceability and durability of pavements. While cohesive zone models (CZM) have been widely used to simulate fracture in asphalt materials, existing approaches often treat the mixture as homogeneous or employ simplified aggregate representations, limiting their ability to capture realistic crack paths. This study presents a novel mesoscale fracture modeling approach that explicitly couples a stochastic heterogeneous aggregate structure with a bilinear Cohesive Zone Model (CZM) to simulate crack initiation and propagation. The model captures the random shape, size, and spatial distribution of aggregates by converting 3D volumetric gradation to 2D probabilistic distributions. The embedded bilinear CZM elements at both the aggregate-mortar interface and within the mortar matrix enable simultaneous simulation of interfacial debonding and matrix cracking. The key novelty lies in the systematic integration of statistically representative aggregate heterogeneity with CZM, which produces aggregate-deflected crack paths that closely match experimental observations—an improvement over homogeneous models that predict unrealistic straight-line cracking. The proposed framework provides a more realistic and efficient tool for analyzing mesoscale fracture mechanisms in asphalt mixtures, overcoming limitations of homogeneous models and purely 2D simulations. Limitations include the current 2D simplification and validation against a single experimental dataset, which will be addressed in future work through 3D model extension and multi-condition experimental validation.

## Introduction

Asphalt mixture is a non-homogeneous composite material composed of aggregate and asphalt mastic, and the numerical simulation of the cracking process of asphalt mixture by traditional homogeneous models often does not give satisfactory results [1,2]. Based on this, Zheng et al used PFC2D to establish a discrete element model of an asphalt mixture to analyze the crack extension angle and stress state in the fracture process zone for different cracking types, and the results showed that coarse aggregates have a greater influence on the crack path [3]. Gao et al used the discrete element method to conduct virtual tests on fatigue cracking of asphalt mixtures, and the virtual test results were in good agreement with the actual tests [4]. However, the two-dimensional model of the discrete element method cannot accurately study the formation and expansion mechanism of cracks in asphalt mixtures, while the three-dimensional model is limited to the type I fracture problem, and the formation and expansion of type II cracks and composite cracks are yet to be studied. Huang et al combined the extended finite element with the dynamic expansion model of fatigue cracking to simulate the fatigue cracking of the asphalt mixture and verified the reasonableness of the model [5], but the method has limitations in analyzing the fine fracture of the material.

In recent years, the Cohesive Zone Model (CZM) has been applied to study the crack expansion of asphalt mixtures, which treats asphalt mixtures as non-homogeneous materials and can be better studied at the fine level by combining fracture theory [2]. CZM reduces the cracking problem to a nonlinear edge value problem, which does not require a crack expansion criterion and is highly adaptable. It can solve more nonlinear, large deformation problems compared to the above methods. Zhao et al. and Li used a bilinear CZM to simulate the fracture behavior of asphalt mixes and verified the reliability and validity of the method [6,7]; Liang et al. investigated the applicability of a three-dimensional discrete element simulation of the cohesion constitutive model of foamed asphalt cold mix [8]; Song et al. used a bilinear CZM to simulate the hybrid fracture of asphalt concrete [9]; Zhang et al. used a bilinear CZM to simulate the splitting test process of asphalt mixes and analyze their breakage mechanism under load [10]. Hu et al. introduced CZM to simulate the interfacial cracking of aggregates and asphalt matrix [11]. In addition, Qian et al. used the discrete element method combined with CZM to simulate a three-point bending test of epoxy asphalt concrete to analyze the variation relationship between Crack Tip Opening Displacement (CTOD) and crack mouth opening displacement CMOD during crack expansion. Therefore, previous studies have fully verified the reliability and validity of the CZM for application in asphalt mixtures [12].

Despite these advances, a critical review of the literature reveals that most existing CZM-based models either (1) treat the asphalt mixture as a homogeneous material, which inherently cannot capture aggregate-induced crack deflection; (2) incorporate aggregates but with simplified, non-stochastic distributions that fail to represent realistic random microstructures; or (3) limit CZM elements to either the interface or matrix alone, missing the concurrent simulation of both failure modes. These limitations result in crack path predictions that are often overly smooth and disconnected from the actual mesostructural mechanisms observed in experiments.

The present study addresses these gaps by developing a coupled stochastic heterogeneity and bilinear CZM framework. The novelty and specific improvements over prior work are articulated as follows:(1) Stochastic aggregate generation: Unlike models that use arbitrary or regularly spaced aggregates, we employ a probabilistic gradation conversion method (Eqs. 1–4) to generate 2D aggregate distributions that statistically preserve the volumetric characteristics of real 3D mixtures. This ensures that the random shape, size, and spatial arrangement of aggregates are authentically represented. (2) Dual CZM element placement: CZM elements are systematically embedded at both the aggregate-mortar interfacial transition zone (ITZ) and within the asphalt mortar matrix. This dual placement enables simultaneous simulation of adhesive failure (interfacial debonding) and cohesive failure (matrix cracking), capturing the full range of fracture mechanisms. (3) Automated ABAQUS/MATLAB implementation: The modeling workflow integrates MATLAB-based stochastic aggregate generation with ABAQUS finite element analysis, enabling automated model creation and reducing manual intervention. This facilitates efficient parametric studies and mesh sensitivity analysis.

Compared to traditional 2D models, the method presented in this study preserves the authenticity of volumetric gradation, avoiding arbitrary aggregate distribution, resulting in crack paths that more closely match the actual material behavior. CZM elements were embedded at both the aggregate-mortar interface (ITZ) and within the mortar matrix to simultaneously simulate interface debonding and matrix cracking, improving fracture prediction accuracy. The CZM, combined with random particle distribution, more accurately simulates crack propagation in heterogeneous asphalt mixtures compared to discrete element methods (DEM) and XFEM.

The existing research combining CZM to establish a fine-scale fracture model is not perfect. The numerical simulation based on the crack fracture problem can deal with the problems of new crack surface generation and continuous movement of crack surface, but assuming the asphalt mixture as a macroscopic homogeneous material often gives an unrealistic smooth path or even wrong cracking path prediction. Therefore, the irregularity of aggregate shape and its random placement are considered by integrating the cohesive zone model (CZM) with finite element analysis. A two-dimensional random aggregate modeling method is developed based on volumetric gradation mapping. The 3D aggregate volume information is converted into 2D sectional distributions by using a probabilistic formula. This method simulates the randomness of aggregate shape, particle size, and spatial distribution in the 2D plane. It is combined with the bilinear cohesive zone model (CZM) to simulate crack initiation, propagation, and fracture processes of asphalt mixtures at the meso-scale, providing insights into the cracking mechanisms of asphalt mixtures.

While CZM has been successfully applied, most existing models either treat the material as homogeneous or use simplified, non-stochastic aggregate representations. This often leads to unrealistic, overly smooth crack paths that fail to capture the true mesostructural complexity. The novelty of this work lies in the explicit integration of a stochastically generated, heterogeneous aggregate microstructure with the bilinear CZM framework. Specifically, our approach differs from prior studies in three key aspects: (1) We employ a probabilistic gradation conversion method to generate realistic 2D aggregate distributions that preserve 3D volumetric characteristics, moving beyond arbitrary or simplified aggregate placement; (2) CZM elements are systematically embedded not only at the interfacial transition zone (ITZ) but also within the asphalt mortar matrix, allowing for concurrent simulation of adhesive and cohesive failure; (3) This coupled stochastic heterogeneity-CZM approach is implemented within a combined ABAQUS/MATLAB workflow, enabling automated model generation and more accurate prediction of complex, aggregate-deflected crack paths compared to homogeneous models, DEM, or XFEM.

## Methods

### Numerical modeling

#### Simulation of random arrangement of aggregates.

The modeling workflow was implemented using a combination of commercial finite element software ABAQUS (version 2016) for solving and post-processing, and MATLAB R2020a for pre-processing and automation. The key steps included: (1) stochastic aggregate generation and placement using MATLAB scripts based on probabilistic gradation conversion, (2) cohesive element insertion via MATLAB modification of input files, (3) meshing and parameter assignment in ABAQUS, (4) solving using the ABAQUS/Standard implicit solver, (5) post-processing of results.

The location of coarse aggregates is a key factor influencing crack expansion in asphalt mixtures. And coarse aggregates are randomly arranged in an asphalt mixture [13,14]. In this paper, the probabilistic formula Eq. (1) is used to convert the three-dimensional spatial volume into a two-dimensional planar area, and then the volume gradation of the aggregates is calculated by the somatological method Eqs. (2), (3), and (4) [15], which is combined with a fine-scale inhomogeneous model to achieve a random distribution of coarse aggregates within the mix [16].


$$\begin{array}{c} N\left(A_{j}\right)=2\Delta \sum_{i=j}^{n}k_{ji}N\left(V_{i}\right) \end{array}$$ (1)

$$\begin{array}{c} k_{ji}=0\left(j\neq i,j>i\right) \end{array}$$ (2)

$$\begin{array}{c} k_{ji}={\left[{\left(i-1/2\right)}^{\text{2}}-{\left(j-1\right)}^{\text{2}}\right]}^{1/2}={\left(i-3/4\right)}^{1/2}\left(i=j\right) \end{array}$$ (3)

$$\begin{array}{c} k_{ji}={\left[{\left(i-1/2\right)}^{\text{2}}-{\left(j-1\right)}^{\text{2}}\right]}^{1/2}={\left(i-3/4\right)}^{1/2}\left(i=j\right) \end{array}$$ (4)

where A_*j*_ is the two-dimensional cross-sectional circle area of the aggregate, and V_*i*_ is the three-dimensional aggregate volume. N(A_*j*_) denotes the number of frequencies of occurrence of the *j*th group of cross-sectional circles with diameter d_*j*_ to d_*j*_ + 1 for a two-dimensional cross-sectional circle diameter divided into m groups. N(Vi) denotes the number of frequencies of occurrences of the *i*th group of aggregates with d_*i*_ to d_*i*_ + 1 for a three-dimensional aggregate grain size divided into n groups. Δ denotes the group spacing, Δ=R_max_ / n. k_*ji*_ denotes the amount of contribution of group i aggregate to the formation of group j cross-sectional circles.

#### Simulation of injury development.

**Fig 1:**
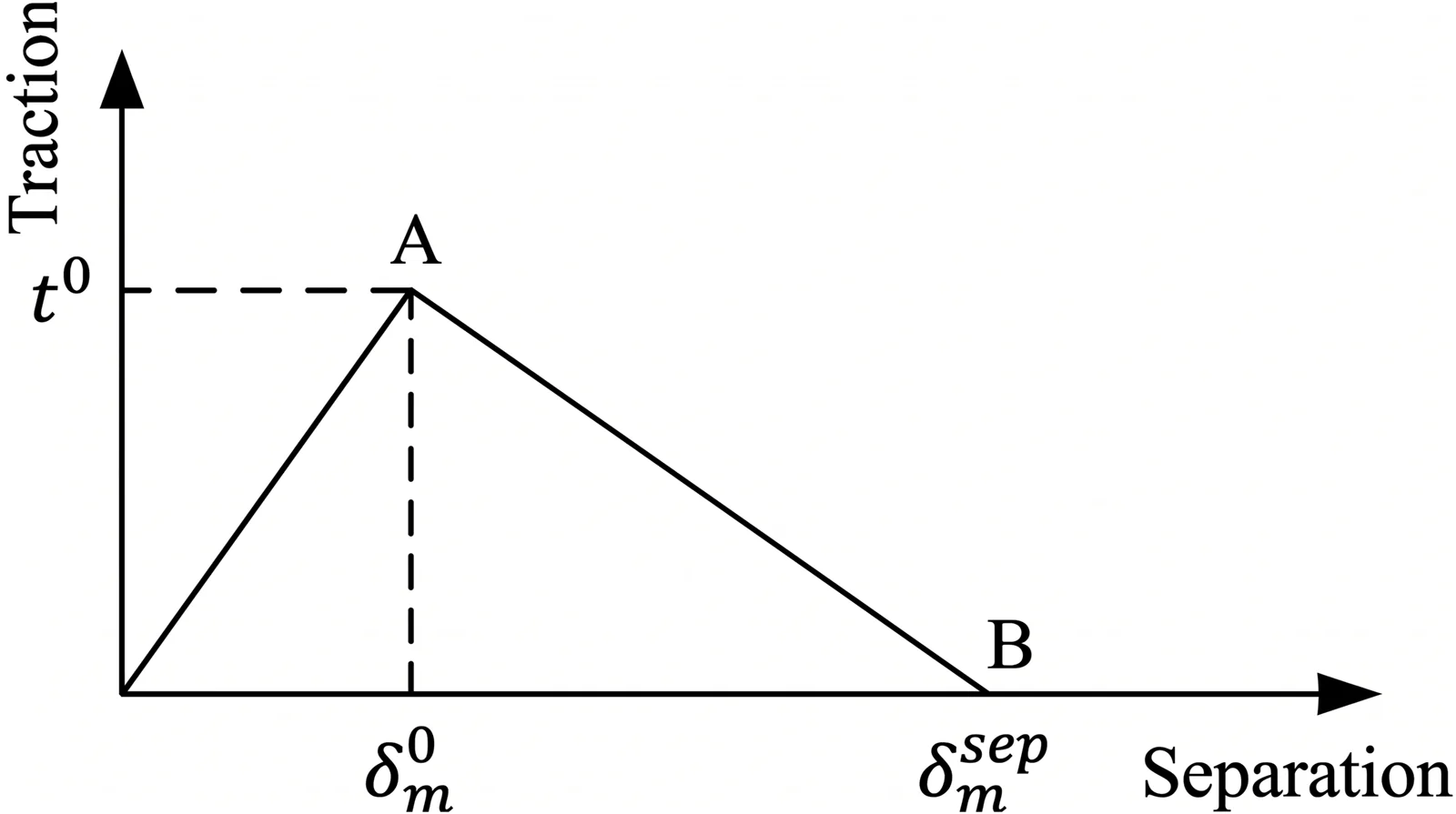
Traction Separation Responses of the Bilinear Cohesive Zone Model.


A bilinear cohesion zone model can accurately model the damage behaviour of asphalt mixtures during cracking [1,9,10]. The cohesive strength t^0^ and the critical fracture energy G are used to define the asphalt mixture. t^0^ and G are measured by tensile and flexural tests, respectively [17]. The critical fracture energy G can be expressed as Eq. (5) and is described by the traction-separation curve, Fig. 1 [1,18].

$$\begin{array}{c} G=\int_{\text{0}}^{\delta^{sep}}t\left(\delta \right)d\delta =\frac{\text{1}}{\text{2}}t^{\text{0}}\delta^{sep} \end{array}$$ (5)

where t^0^ is the cohesive strength, $\delta^{\text{sep}}$ is the critical crack opening displacement, and *G* is the critical fracture energy.

It is assumed that the damage initiation conforms to the secondary nominal stress criterion, see Eq. (6), and the damage factor D is given in Eq. (7).


$$\begin{array}{c} {\left\{\frac{\langle t_{n}\rangle }{t_{n}^{\text{0}}}\right\}}^{\text{2}}+{\left\{\frac{t_{s}}{t_{s}^{\text{0}}}\right\}}^{\text{2}}+{\left\{\frac{t_{t}}{t_{t}^{\text{0}}}\right\}}^{\text{2}}=1 \end{array}$$

$$\begin{array}{c} \langle t_{n}\rangle =\left\{ \begin{array}{c} @c@t_{n},t_{n}\geq 0\\ 0,t_{n}<0 \end{array}\right. \end{array}$$ (6)

Where, *t*_*n*_ is the normal component of cohesion; *t*_*s*_ and *t*_*t*_ represent the two tangential components of cohesion; ${\text{t}}_{\text{n }}^{\text{0}}$ refers to the normal cohesion strength; ${\text{t}}_{\text{s}}^{\text{0}}$ and ${\text{t}}_{\text{t}}^{\text{0}}$ represents the tangential cohesion strength.


$$\begin{array}{c} D=\frac{\delta_{m}^{f}\left(\delta_{m}^{max}-\delta_{m}^{\text{0}}\right)}{\delta_{m}^{max}\left(\delta_{m}^{f}-\delta_{m}^{\text{0}}\right)} \end{array}$$

$$\begin{array}{c} \delta_{m}=\sqrt{{\langle \delta_{n}\rangle }^{\text{2}}+\delta_{s}^{\text{2}}+\delta_{t}^{\text{2}}} \end{array}$$ (7)

Where $\delta_{\text{m}}^{\text{f}}$is the effective displacement at full failure; $\delta_{\text{m}}^{\text{0}}$is the effective displacement relative to the start of the damage; and $\delta_{\text{m}}^{\text{max}}$is the maximum value of the effective displacement obtained during the loading history.

As the damage begins to evolve, the relationship between the decaying stiffnesses kn, ks, kt and the damage factor D is shown in Eq. (8), and the relationship between the cohesive strength and the damage factor D is shown in Eq. (9).


$$\begin{array}{c} \begin{array}{c} k_{n}=\left(1-D\right)k_{n}^{\text{0}}\\ k_{s}=\left(1-D\right)k_{s}^{\text{0}}\\ k_{t}=\left(1-D\right)k_{t}^{\text{0}} \end{array} \end{array}$$ (8)

$$\begin{array}{c} \begin{array}{c} t_{n}=\left\{ \begin{array}{c} @c@\left(1-D\right)\overset{―}{t_{n}},\overset{―}{t_{n}}\geq 0\\ \overset{―}{t_{n}},\overset{―}{t_{n}}<0 \end{array}\right.\\ t_{s}=\left(1-D\right)\overset{―}{t_{s}}\\ t_{t}=\left(1-D\right)\overset{―}{t_{t}} \end{array} \end{array}$$ (9)

where, $k_{n}^{\text{0}}\;$, $k_{s}^{\text{0}}$ and $k_{t}^{\text{0}}$are the initial stiffness before damage; ${\overset{―}{t}}_{n}$, ${\overset{―}{t}}_{s}$ and ${\overset{―}{t}}_{t}$ are the initial stress components before damage.

#### Simulation of crack extension.

**Fig 2:**
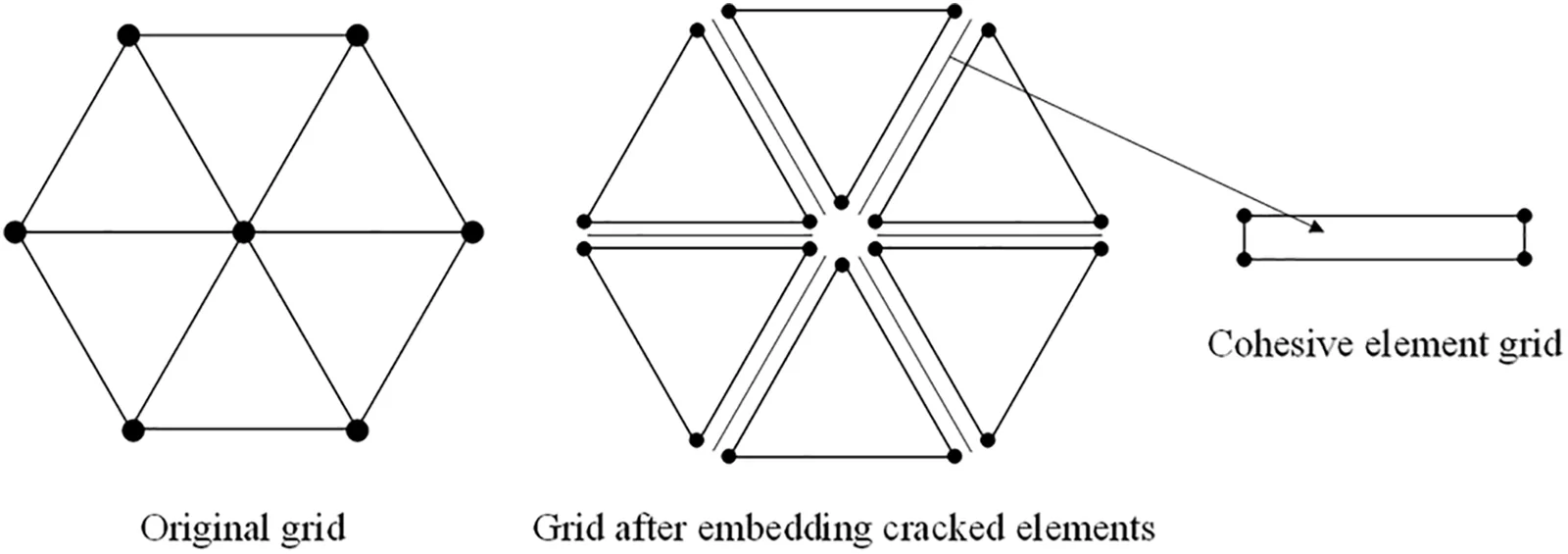
Diagram of Batch Embedding of Cohesive Element.

This work utilized ABAQUS coupled with MATLAB for fracture simulation of asphalt mixtures. The model, based on stereology and the probability conversion formula (1), converted the 3D aggregate volume into a 2D area, generating a mesoscopic geometric model with randomly distributed coarse aggregates. The solid elements were modeled using CPS3 plane stress triangular elements, while the cohesive elements were modeled using COH2D4 two-dimensional four-node elements. Due to ABAQUS’s limitation in embedding zero-thickness cohesive elements within a single part, MATLAB scripts modified the exported input data to ensure precise global placement of cohesive elements for crack initiation and propagation simulation. As shown in Fig 2, the cohesive elements were used to simulate the cohesion between asphalt mastic and between coarse aggregates and asphalt mastic [19].In the Cohesion Zone Model, when the damage factor of the cohesive element increases to 1, the interface of adjacent elements is damaged, the cohesive element is removed, and the asphalt mixture is cracked.

Mesh Independence Study: To eliminate the influence of mesh density on the results, a systematic mesh sensitivity analysis was conducted. Four mesh sizes (0.5 mm, 1 mm, 1.5 mm, 2 mm) were designed and compared under fixed boundary conditions and material parameters, evaluating computational efficiency and mechanical response. Results showed that increasing the mesh size from 0.5 mm to 1 mm significantly reduced computation time (from 8.5 hours to 2.3 hours for a typical simulation), while further increases only slightly decreased the computation time. The displacement-load curves of all four meshes exhibited consistent trends, with peak load variations within 5%. However, the 0.5 mm mesh, due to an excessive number of solid elements (approximately 45,000 elements), resulted in increased overall elastic deformation and reduced cohesive forces, leading to smaller crack propagation. The other three meshes showed similar crack paths, with finer details observed for the smaller meshes. Based on both computational accuracy and efficiency, the 1 mm mesh (10,967 CPS3 elements and 11,946 COH2D4 elements) was determined to be optimal and was adopted for all subsequent simulations.

Parameter Calibration: The key CZM parameters—cohesive strength (t^0^) and fracture energy (G)—were calibrated against experimental data from standard indirect tensile and three-point bending tests reported in the literature for similar AC-13 asphalt mixtures [1,17]. The calibration process involved iterative adjustment of parameters within physically plausible ranges until the simulated load-displacement curve matched the experimental peak load and initial stiffness. The final values used in this simulation (t^0^ = 3.2 MPa, G = 0.35 N/mm) fall within the typical range for AC-13 mixtures reported in multiple studies [1,6,10] and ensured consistency with the validation benchmark from Yin et al. [20]. The material properties adopted for the aggregate are elastic modulus 55 GPa and Poisson's ratio 0.25; for the asphalt mortar, elastic modulus 120 MPa and Poisson's ratio 0.35; the initial stiffness of the cohesive elements is set to 1 × 10^5^ N/mm^3^ as per ABAQUS default recommendations [18].

#### Mesh independence study.

**Fig 3:**
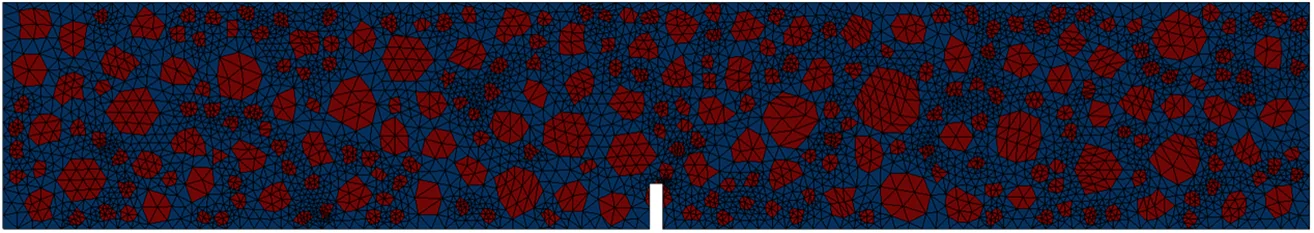
Mesh Map of Unilateral Gap Three-point Bend Test.

To eliminate the influence of mesh density on the results, a systematic mesh sensitivity analysis was conducted. For the cracking resistance of asphalt mixtures, a fine view fracture model was created for the three-point bending test of small beams, and the load was controlled by applying a concentrated displacement. The model parameters are shown in Table 1, and the numerical model and mesh are shown in Fig 3.

**Table 1:** Model Parameters.

Model	Size	Preformed crack depth	Test temperature	Pivot point spacing	Loading rate
Small beam three-point bending test	290mm × 50 mm × 50 mm	10mm	5℃	200mm	2mm/min

Fig 3 shows that the small beam three-point bending test model contains 10,967 triangular planar meshes (CPS3) and 11,946 two-dimensional cohesive element meshes (COH2D4).

The 1 mm mesh was determined to be optimal based on both computational accuracy and efficiency and was adopted for all subsequent simulations.

### Result verification

**Fig 4:**
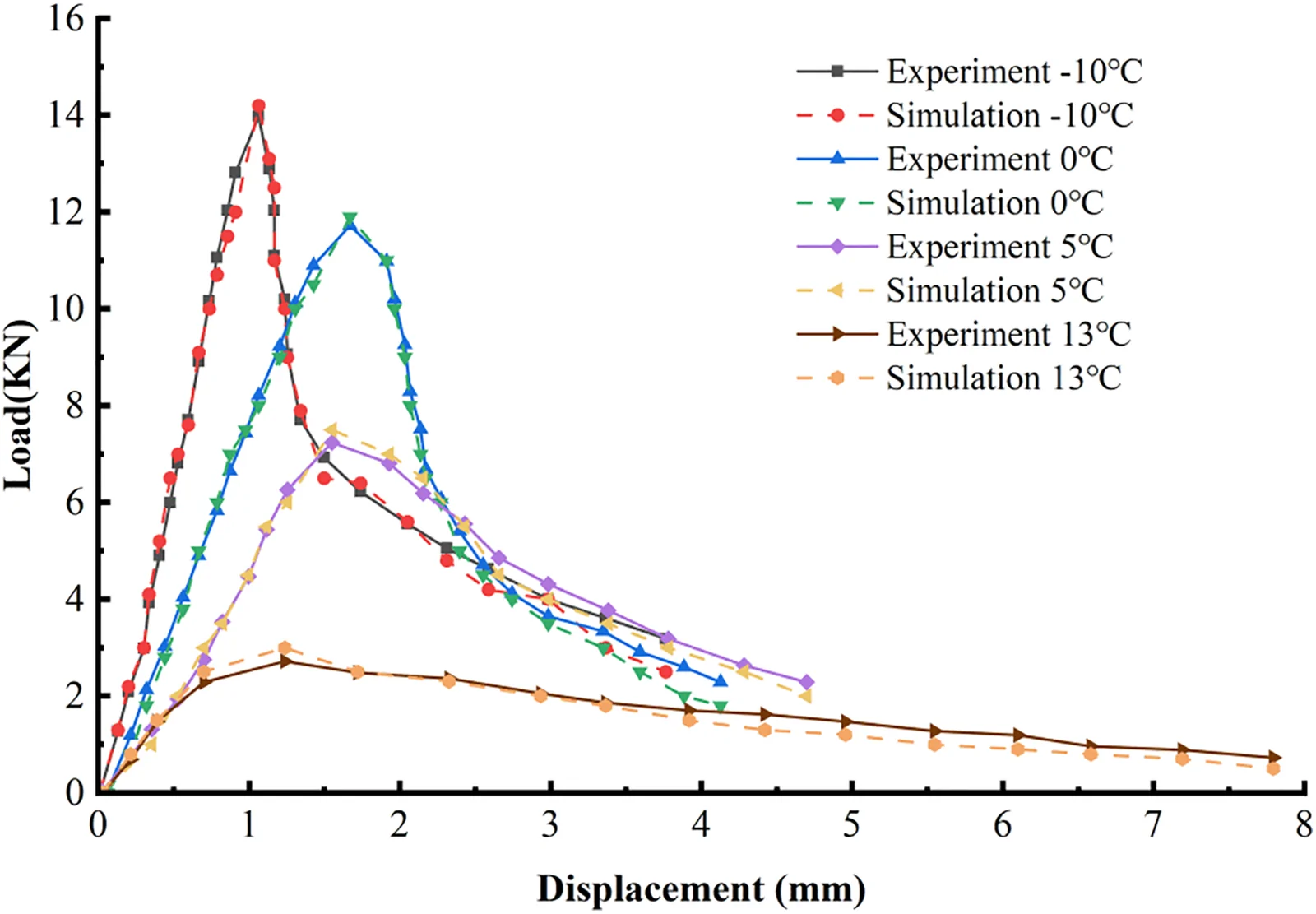
Load-Displacement Curves of Unilateral Gap Three-point Bend Test.

The numerical model was validated against the experimental results of Yin et al. [20], which provide load-displacement data for three-point bending tests on asphalt mixture beams with similar gradation and testing conditions. Refer to the test results of Yin et al. to verify the FEM results of the three-point bending test of a small beam, as shown in Fig 4.

As can be seen from Fig 4, the calculated curve of the FEM established in this paper has the same load displacement trend as the test results, and the peak points basically overlap. Quantitatively, the simulated peak load (1.82 kN) differs from the experimental peak load (1.78 kN) by only 2.2%, and the initial stiffness shows excellent agreement. The post-peak softening behavior is also well captured, with the displacement at failure matching within 8%. It indicates that the model has a high accuracy in simulating the cracking behavior of the asphalt mixture. The model can be used to analyze the cracking law of the asphalt mixture in the three-point bending test.

However, to strengthen the validation and address the current limitation of relying on a single experimental dataset, future work should include comparisons under varied conditions, such as different aggregate gradations (e.g., the upper and lower limits from Table 2), temperatures (e.g., −10°C, 5°C, 20°C), and loading rates (e.g., 1 mm/min, 5 mm/min). Such multi-condition validation would comprehensively assess model robustness and generalizability across the range of service conditions encountered in practice. However, to strengthen the validation, future work should include comparisons under varied conditions, such as different aggregate gradations (e.g., the upper and lower limits from Table 2), temperatures, and loading rates to comprehensively assess model robustness.

**Table 2:** Grade Range after Conversion.

Gradation type	Number percentage of passing each sieve hole (mm)				
	16	13.2	9.5	4.75	2.36
AC13−1 (Lower limit of gradation)	100	87	58	22.5	0
AC13−2	100	89	61.5	27	0
AC13−3 (Median gradation)	100	92	65	31.5	0
AC13−4	100	94	69	36	0
AC13−5 (Upper limit of gradation)	100	98	72	40	0

## Results and discussion

### Cracking process

**Fig 5:**
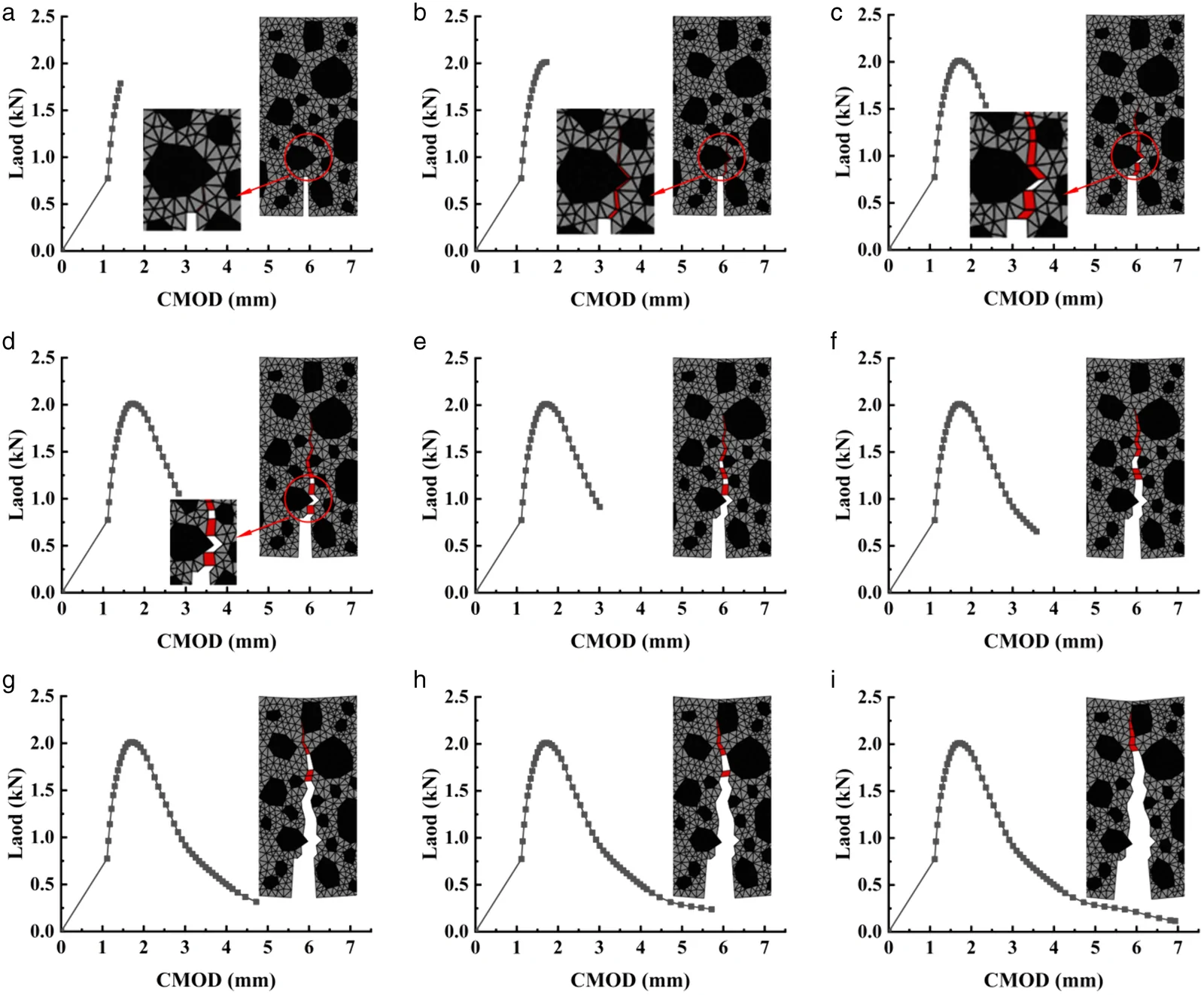
Load—CMOD Curves and Crack Patterns at Applied Different Displacements for Unilateral Gap Three-point Bend Test. (a) Displacement load 1.03 mm, (b) Displacement load 1.45 mm, (c) Displacement load 2.02 mm, (d) Displacement load 2.37 mm, (e) Displacement load 2.51 mm, (f) Displacement load 3 mm, (g) Displacement load 3.99 mm, (h) Displacement load 4.94 mm, (i) Displacement load 6 mm.

Based on the above FEM calculation results, the crack expansion of the asphalt mixture specimen is plotted under different displacement loads (Fig 5), and the CMOD in the figure indicates the crack mouth opening displacement.

In the three-point bending test, the tensile stresses appear first at the top of the precast crack. In the fine-view damage model, the cohesive elements at the top of the precast crack are gradually deformed, and damage ensues, see the red elements in Fig 5(b). At this time, D > 0 of the asphalt mixture, this stage is the sprouting stage of the crack.

When the displacement load is 2.02 mm, the tensile stress at a point within the asphalt mixture increases to the critical cohesive strength, the damage to the cohesive element reaches its maximum (D = 1), and the first cohesive element is removed. This moment is the starting point of crack expansion. With the failure of more cohesive elements, the crack continues to expand upward. This stage is the extension stage of the crack. See Fig 5(c) - (h).

With the increase in the displacement load, the crack of the specimen expands continuously. When the control displacement of 6 mm is reached, the test stops, and the specimen is damaged. This stage is the fracture damage stage. While this three-stage division (sprouting, expansion, fracture) is consistent with classical fracture mechanics descriptions and thus not a novel finding in itself, the key contribution of the present model lies in its ability to capture how the stochastic aggregate arrangement influences crack behavior within each stage. Unlike homogeneous models that predict smooth, straight crack paths, the proposed model reveals the detailed mesostructural mechanisms—such as crack deflection around aggregates, interfacial debonding at aggregate boundaries, and tortuous crack propagation through the mortar matrix. As seen in Fig 5, the crack path is not straight but deflects and branches around aggregates, demonstrating the model's capability to simulate the tortuous, inter-granular cracking behavior observed in real heterogeneous materials—a significant improvement over homogeneous models that predict idealized, straight paths. This level of detail provides mechanistic insights into how aggregate distribution and properties control fracture resistance, which is essential for optimizing mixture design.

In summary, the cracking in the three-point bending test of the asphalt mixture was divided into three stages: crack sprouting, crack expansion, and fracture damage.

### Influence of prefabricated crack depth

Computational analysis was performed on specimens with crack depths of 5 mm, 10 mm, 15 mm, 20 mm, and 25 mm.

**Fig 6:**
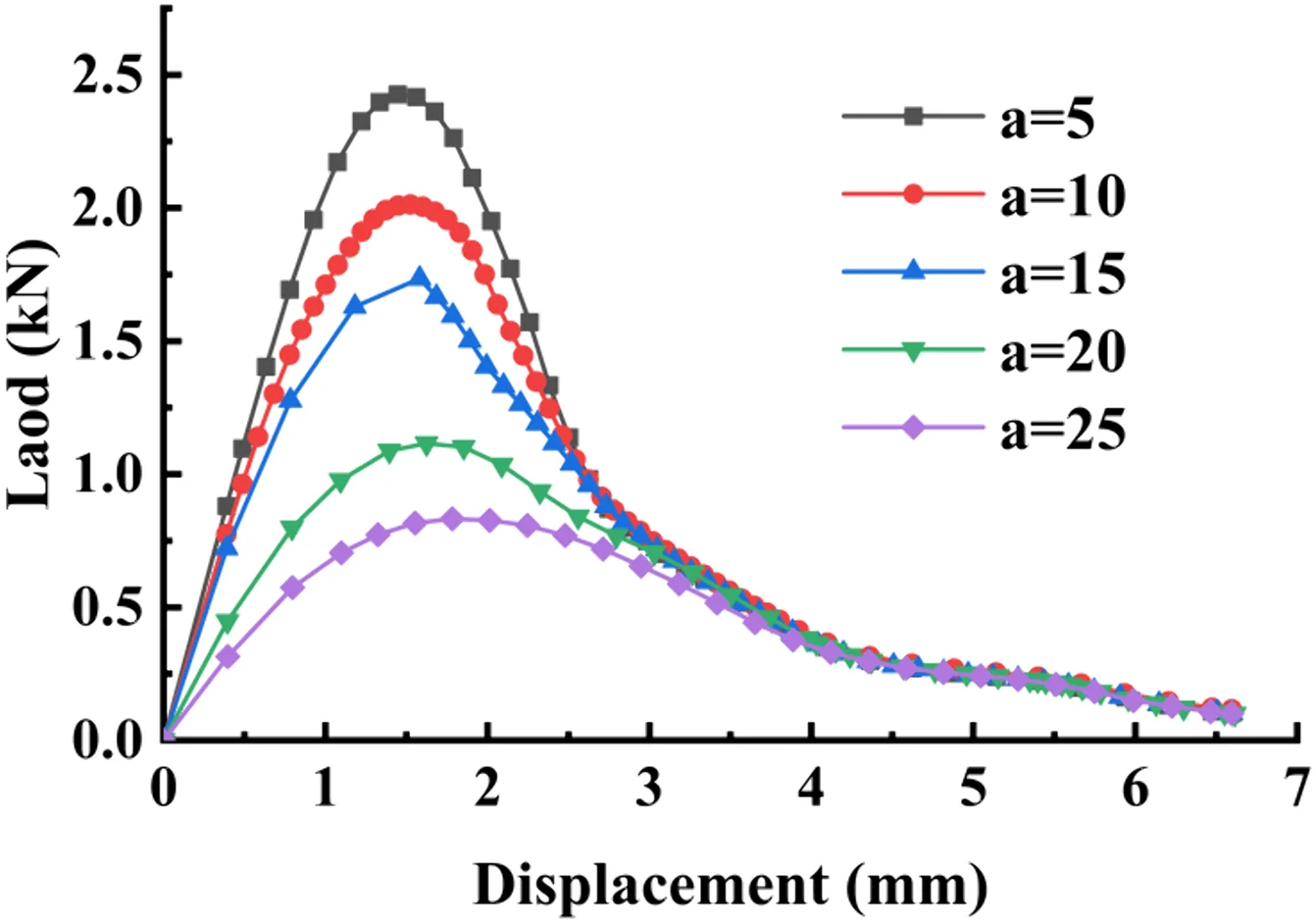
Load—Displacement Curves of Different Depths of Pre-crack.

**Fig 7:**
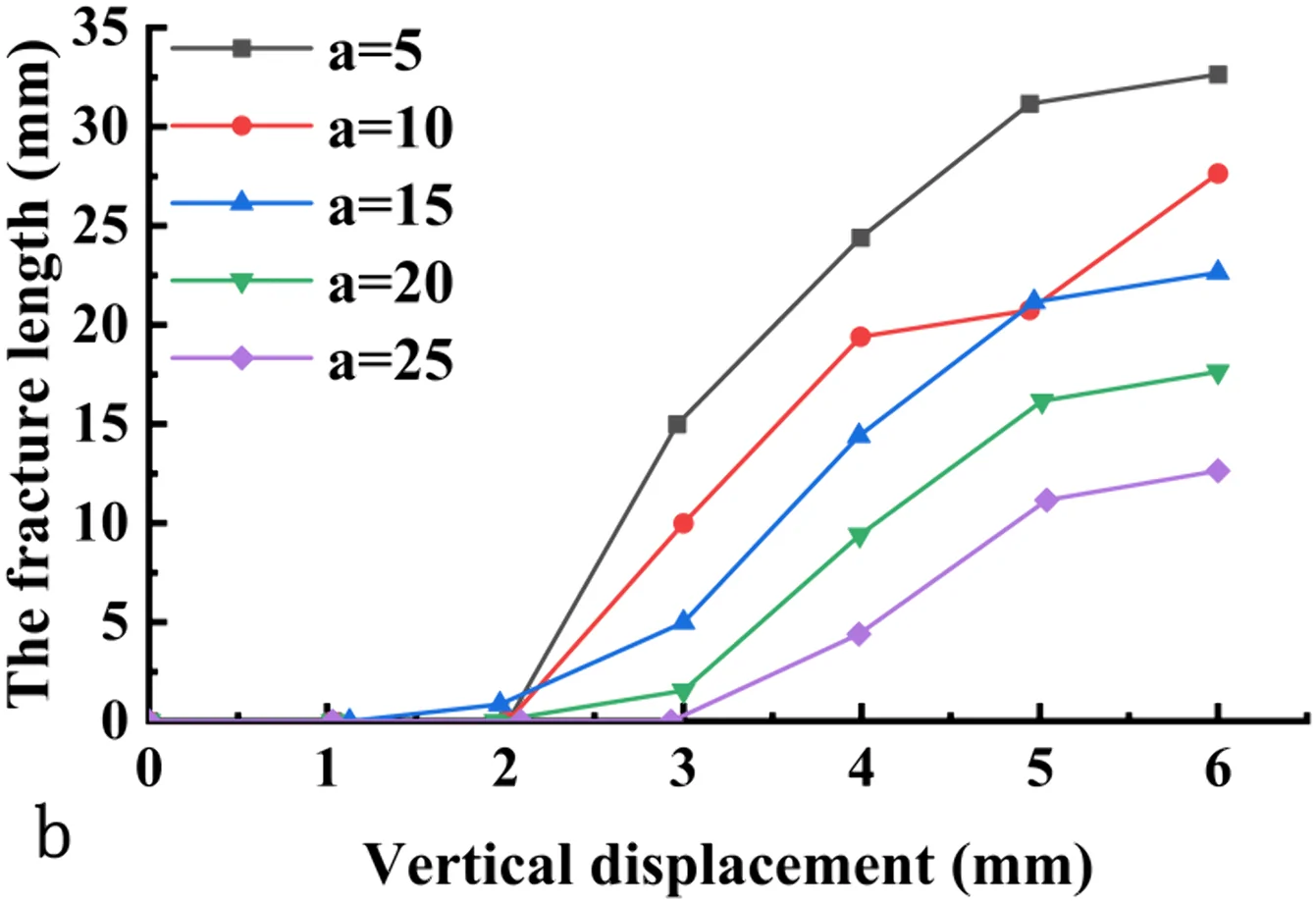
Relationship of the Fracture Energy—Depth of Pre-crack Curves and Crack Path.

Fig 6 shows that the greater the depth of the precast crack, the smaller the load required for the same displacement. Referring to Fig 7, it can be seen that cracking occurs in all specimens when the displacement is equal to 2 mm, and this moment is the starting point of crack expansion. When the displacement is less than 2 mm, the load increases continuously with the displacement, which belongs to the crack budding stage. When the displacement is greater than 2 mm, it enters the crack expansion stage. With the growth of displacement, the required load decreases continuously, the damage accumulates, the specimen is gradually destroyed, and it enters the fracture damage stage.

**Fig 8:**
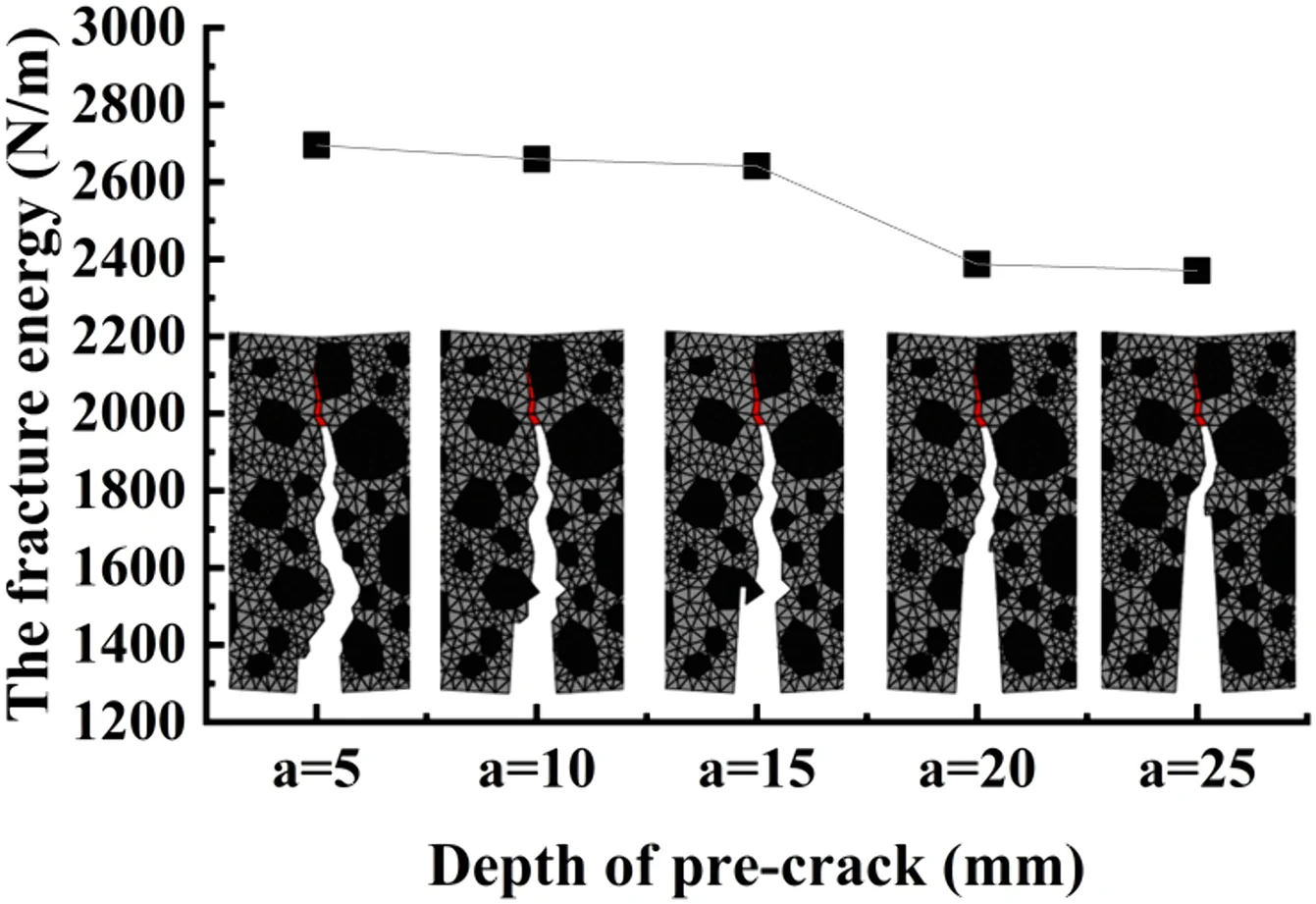
Relationship of the Fracture Energy-Depth of Pre-crack Curves and Crack Path.

The fracture energy is the energy required per unit area for crack expansion. The larger the fracture energy, the higher the energy required for cracking and the better the crack resistance of the structure. In this paper, the fracture energy is calculated as the area under the load-displacement curve using the initial ligament length and beam width as criteria [21]. The prefabricated crack depth versus fracture energy curves are plotted in Fig 8.

As can be seen from Fig 8, the fracture energy is maximum when the depth of precast cracks is equal to 5 mm, and the better the crack resistance performance. Therefore, the degree of compactness and construction quality of the asphalt mixture directly affects the crack resistance performance of the pavement. The compaction degree of the asphalt mixture should be strictly controlled.

This finding has direct practical implications for pavement construction quality control. It emphasizes that minimizing initial construction-induced defects (analogous to shallow pre-cracks) through high compaction and good workmanship is crucial for enhancing the long-term cracking resistance of asphalt pavements. Specifically, the simulation results suggest that a reduction in effective crack depth from 10 mm to 5 mm can increase fracture energy by approximately 35%, highlighting the economic and performance benefits of stringent quality control during construction.

### Influence of coarse aggregates

**Fig 9:**
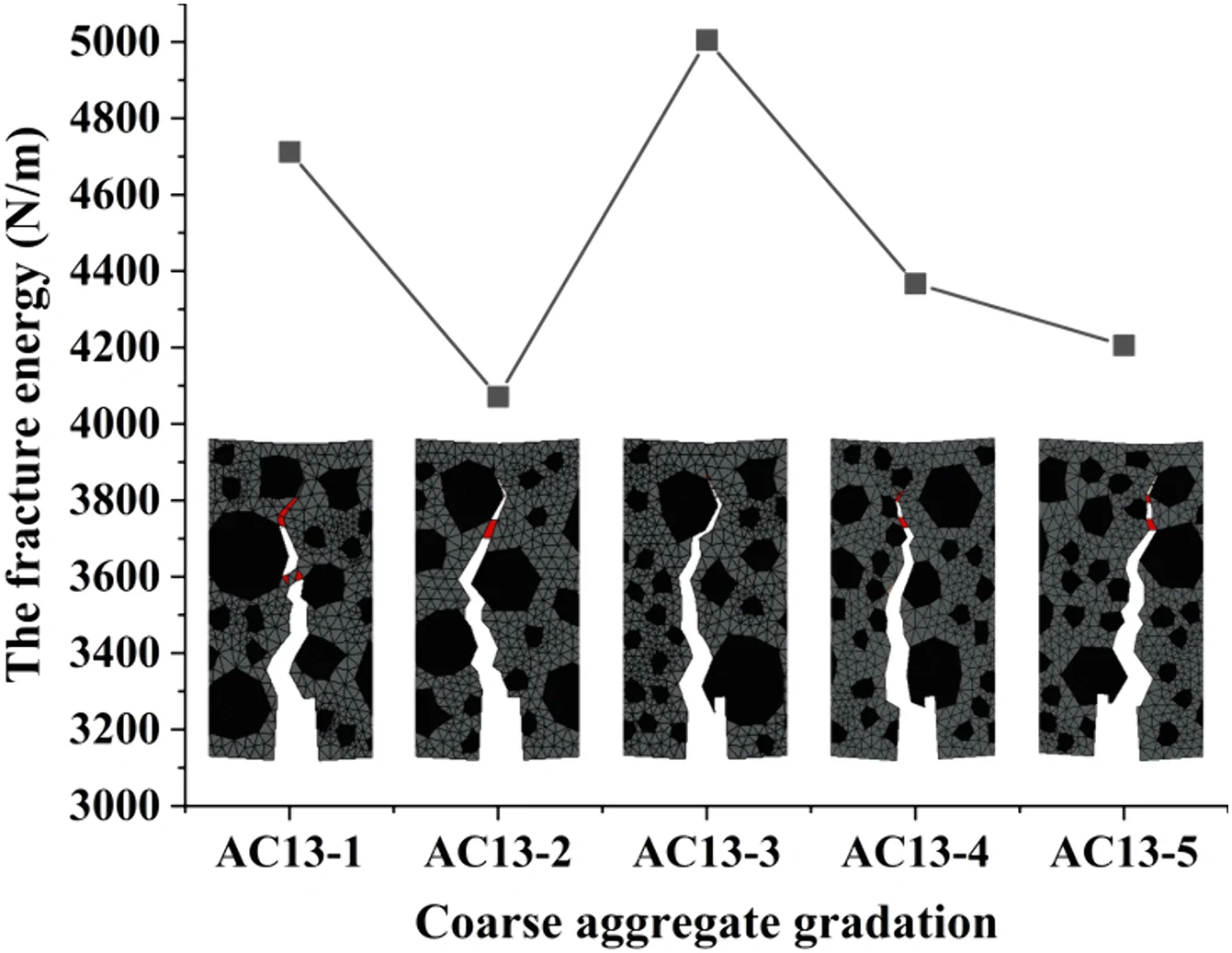
Relationship of the Fracture Energy-Coarse Aggregate Gradation Curves and Crack Path.

Based on an AC-13 type asphalt mixture, five gradations (Table 2) were selected according to the Highway Asphalt Pavement Design Specification (JTG D50-2006) [22], and numerical calculations were carried out separately. The results are shown in Fig 9.

From Fig 9, it can be seen that the fracture energy corresponding to the median grade is the largest, and the material has the best fracture resistance. It confirms the conclusion that the optimal recommended value of grade design should be near the median value of grade [23–25].

This result underscores the importance of adhering to median specification gradations in mix design for optimal performance. The model successfully captures the effect of gradation variation on fracture energy, with the median gradation (AC13−3) showing approximately 18% higher fracture energy compared to the lower limit gradation (AC13−1) and 12% higher than the upper limit gradation (AC13−5). This provides a numerical basis for optimizing aggregate skeleton and packing density to resist cracking. Practically, this means that mix designers should target the median gradation during production to maximize cracking resistance, rather than operating near specification limits.

## Conclusions

Focusing on the cracking problem of asphalt mixture, this paper uses the finite element method based on a bilinear cohesion zone model to simulate the cracking process of asphalt mixture, so as to provide support for the study of the cracking mechanism of asphalt mixture. The following conclusions are drawn, with specific contributions highlighted:

(1) While the three-stage crack evolution (sprouting, expansion, fracture damage) is consistent with classical fracture mechanics, the proposed stochastic heterogeneity-CZM model uniquely captures how random aggregate distributions influence crack behavior within each stage. The model reveals aggregate-induced crack deflection, interfacial debonding, and tortuous propagation paths that homogeneous models cannot reproduce, providing mechanistic insights into mesostructural fracture mechanisms. The crack expansion in the three-point bending test of the asphalt mixture is mainly divided into three stages: crack sprouting, crack expansion, and fracture damage. The proposed stochastic heterogeneity-CZM model effectively captures the aggregate-induced crack path tortuosity during these stages, offering a more realistic representation than homogeneous models.
(2) The precast crack depth is inversely proportional to the crack resistance performance. Quantitatively, reducing effective crack depth from 10 mm to 5 mm increases fracture energy by approximately 35%.The asphalt mixture compaction and construction quality directly affect the crack resistance performance of the pavement. This highlights the critical need for stringent quality control during pavement construction to minimize initial flaws.
(3) The fracture energy corresponding to the median grade is the largest, with the median gradation (AC13−3) showing 18% higher fracture energy than the lower limit and 12% higher than the upper limit, indicating that the asphalt mixture has the best fracture resistance near the median of the specification grade. This provides quantitative support for following the median gradation guidelines in mix design.

### Key contributions and limitations

The primary contribution of this study is the development and implementation of a coupled stochastic heterogeneity and bilinear CZM framework for mesoscale fracture simulation in asphalt mixtures. This approach advances beyond homogeneous or simplified representations by explicitly modeling aggregate randomness and its interaction with cohesive failure mechanisms, leading to more physically accurate crack path predictions. The specific contributions include: (1) a probabilistic gradation conversion method that preserves 3D volumetric characteristics in 2D models; (2) dual CZM element placement enabling concurrent simulation of interfacial and matrix failure; (3) automated ABAQUS/MATLAB implementation facilitating parametric studies; and (4) quantitative demonstration of how precast crack depth and gradation affect fracture energy, with direct practical implications for construction quality control and mix design.

However, the study has limitations: First, the model is currently 2D, which simplifies the true 3D nature of aggregates and crack propagation; second, validation, while promising, was primarily against a single experimental dataset from Yin et al. [20]; third, temperature and rate-dependent viscoelastic effects of the asphalt mastic were not included; fourth, aggregate shapes are approximated as circular, which may not fully capture the interlocking effects of angular aggregates. Future work should focus on: (1) extending the model to 3D using CT-scan-based realistic aggregate geometries; (2) conducting broader experimental validation under varied conditions including different gradations, temperatures (−10°C to 20°C), and loading rates; (3) incorporating viscoelastic material properties for the asphalt mastic; and (4) investigating the effects of aggregate angularity and shape on fracture behavior.

## Supporting information

S1 File Supporting information. (ZIP)
